# Respiratory Syncytial Virus Vaccines: A Review of the Candidates and the Approved Vaccines

**DOI:** 10.3390/pathogens12101259

**Published:** 2023-10-19

**Authors:** Xanthippi Topalidou, Alexis M. Kalergis, Georgios Papazisis

**Affiliations:** 1 Department of Clinical Pharmacology, School of Medicine, Aristotle University of Thessaloniki, 54124 Thessaloniki, Greece; xanthippitopalidou@gmail.com; 2 Millennium Institute of Immunology and Immunotherapy, Facultad de Ciencias Biológicas, Pontificia Universidad Católica de Chile, Santiago 8320000, Chile; akalergis@bio.puc.cl; 3 Departamento de Endocrinología, Facultad de Medicina, Pontificia Universidad Católica de Chile, Santiago 8320000, Chile; 4 Clinical Research Unit, Special Unit for Biomedical Research and Education, School of Medicine, Aristotle University of Thessaloniki, 54124 Thessaloniki, Greece

**Keywords:** respiratory syncytial virus (RSV), vaccine development, prevention, lower respiratory tract infection (LRTI), vaccine candidates, subunit vaccine, live-attenuated vaccine, mRNA vaccine, recombinant vaccine

## Abstract

Respiratory syncytial virus (RSV) is responsible for a significant proportion of global morbidity and mortality affecting young children and older adults. In the aftermath of formalin-inactivated RSV vaccine development, the effort to develop an immunizing agent was carefully guided by epidemiologic and pathophysiological evidence of the virus, including various vaccine technologies. The pipeline of RSV vaccine development includes messenger ribonucleic acid (mRNA), live-attenuated (LAV), subunit, and recombinant vector-based vaccine candidates targeting different virus proteins. The availability of vaccine candidates of various technologies enables adjustment to the individualized needs of each vulnerable age group. Arexvy^®^ (GSK), followed by Abrysvo^®^ (Pfizer), is the first vaccine available for market use as an immunizing agent to prevent lower respiratory tract disease in older adults. Abrysvo is additionally indicated for the passive immunization of infants by maternal administration during pregnancy. This review presents the RSV vaccine pipeline, analyzing the results of clinical trials. The key features of each vaccine technology are also mentioned. Currently, 24 vaccines are in the clinical stage of development, including the 2 licensed vaccines. Research in the field of RSV vaccination, including the pharmacovigilance methods of already approved vaccines, promotes the achievement of successful prevention.

## 1. Introduction

RSV is an enveloped, negative-sense, single-stranded RNA virus causing seasonal infections in a biphasic age distribution, affecting more frequently children until the age of 2 years with a higher frequency across the age spectrum from 6 weeks to 6 months, as well as older adults due to the reduction of immunity [1,2,3]. The subtypes of RSV named RSV Type A (RSV-A) and RSV Type B (RSV-B) cause infection throughout the season, with one of the two prevailing each year [4]. Infancy under 3 months of age, prematurity or cardiopulmonary diseases in the pediatric population, and aging or medical immunosuppression in adult patients are defined risk factors linked proportionally to the severity of the disease [2]. The clinical manifestation of the infection is, in most cases, an upper respiratory tract disease, which can further develop into a lower respiratory tract disease, especially in patients with risk factors [5]. In young children, bronchiolitis, pneumonia, and tracheobronchitis are manifestations of RSV infection [6]. Compared to the younger adult population, which usually experiences upper respiratory tract symptoms, older adults and immunocompromised adults experience more severe manifestations of the infection, including bronchiolitis, pneumonia, and asthma, worsening of chronic obstructive pulmonary disease (COPD), or congestive heart failure exacerbations [7]. 

Therapeutic interventions are limited to patient support to maintain the hydration and nutrition status at adequate levels, decongestion from secretions, adequate oxygen supply, and mechanical ventilation in severe cases [8,9]. Prevention strategies include precautionary measures to avoid RSV exposure and passive and active immunization [8]. Passive immunization using monoclonal antibodies is a method used for high-risk infants. A monoclonal antibody named Palivizumab is a prophylactic drug used in infants susceptible to severe infection [10,11]. Beyfortus^®^ (Nirsevimab) is also a monoclonal antibody with improved activity compared to Palivizumab. The drug was approved for market use by the European Medicines Agency (EMA) in all infants during the first RSV season for the protection against RSV lower respiratory tract infection (LRTI) in November 2022 [10,12,13,14].

Eleven structural and non-structural proteins constitute the virus, of which the fusion (F) glycoprotein, attachment (G) glycoprotein, and small hydrophobic (SH) proteins are surface proteins on the lipid bilayer outer membrane. The F protein plays a key role in the fusion process of the cell with the viral membranes and is also of secondary importance for attachment, a process mediated through the glycosylated G protein. The G protein consists various domains fluctuating between the different RSV isolates. An exception is a specific site at the central domain of the G protein, which remains stable. In contrast, the F protein is more conserved among serotypes [15,16]. The SH protein is thought to participate in the virus’s replication process and extension of the period until apoptosis. Thus, the removal of the SH protein results in viral attenuation [16]. The remaining proteins include M2-1 and M2-2 regulating the transcription process, the non-structural proteins NS1 and NS2 acting against apoptosis and interferon (IFN)-mediated responses, Matrix (M) supporting the envelope in contact with the nucleoprotein (N), and the RNA-dependent RNA polymerase complex (RdRp) containing the long polymerase subunit (L), a phosphoprotein polymerase cofactor (P), and N, both of which help in virus transcription [3,15,17].

Active immunization is mainly aimed at specific vulnerable populations. Specifically, immunization strategies target infant, children, maternal, and older adult populations, due to high susceptibility rates. Furthermore, immunization techniques are adapted to the needs and specific characteristics of each of the aforementioned target groups. Historically, the development of RSV vaccines was hampered for years as scientists intervened, concerned about the possible occurrence of enhanced respiratory disease (ERD) after the unsuccessful formalin-inactivated RSV vaccine, which was tested in the 1960s, resulting in ERD in the majority of the seronegative participants after RSV exposure and two cases of death. T-helper 2 (Th2) immunity was associated with the clinical manifestation of the disease [18,19]. 

The different types of developed RSV vaccines include mRNA, subunit and particle-based, live-attenuated or chimeric vaccines, and recombinant-vector-based vaccines. During the period of the severe acute respiratory syndrome coronavirus 2 (SARS-CoV-2) pandemic, mRNA vaccines were manufactured and licensed for market use in a short period of time, constituting a source of information regarding adverse events (AEs) through large-scale pharmacovigilance. High levels of immunity induction can be reached without invading the genome of the recipient, providing a good safety profile [20]. Subunit vaccines are manufactured with the addition of adjuvants to improve the presentation of the antigen to the host cells and strengthen the responses of the host. This category is not preferred for the development of RSV vaccines in the pediatric population due to the possibility of ERD development. Several subunit vaccine candidates are F-protein-based [21]. Live-attenuated vaccines represent an appropriate candidate for the pediatric population not exposed to RSV because a linkage to ERD development is weak, and they provide a painless vaccination method, due to intranasal delivery [22]. Chimeric vaccines overcome the difficulty of achieving long-term immunity in the case of RSV since the desired antigens are inserted into another potent virus, which induces immunity for longer periods of time [23]. The use of a vector-based vaccine can be applied to pediatric and older adult populations without infection risk, enhancing the response through the vectors’ characteristics [24].

The present narrative review aims to provide an overview of the registered clinical trials of the candidate and the recently approved RSV vaccines.

## 2. Materials and Methods

The present paper is a narrative review of the published literature in the field of active immunization agents against RSV. Reports and the analysis of the registered clinical trials of vaccine candidates and already approved vaccines are presented. The review included only vaccine candidates in the clinical development phase at the time of conducting the research, with the last update being on 24 August 2023. Preclinical vaccine candidates, as well as immunoprophylaxis drugs, were not reviewed. RSV Vaccine and monoclonal antibody (mAb) Snapshot from PATH (Center for Vaccine Innovation and Access) was used for the verification of the current vaccine candidates (last update: July 2023) [25]. The Cochrane Database, MEDLINE, and EMBASE databases were researched, while the research of clinical trial registries, the databases of the EMA and the United States Food and Drug Administration (FDA), and the websites of all the pharmaceutical companies involved in RSV vaccine development was conducted separately. Language restriction was applied, including only English bibliography without date or further restrictions. General information about the virus and the vaccine categories of the current RSV vaccine candidates were reviewed and presented in the report’s introduction.

## 3. Results

**Figure 1:**
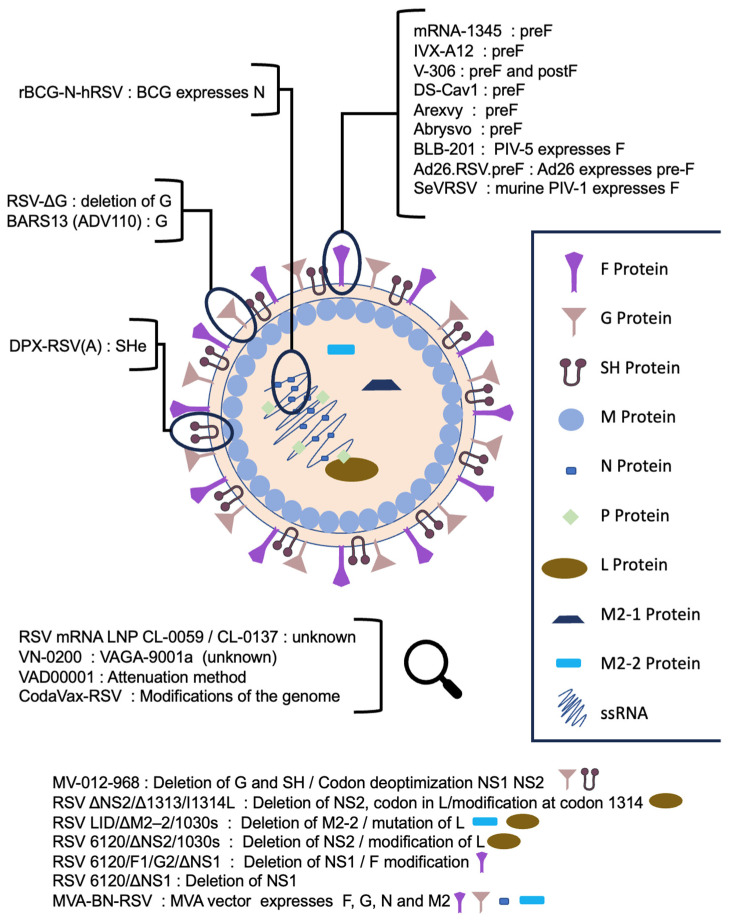
Structure of the RSV virion and categorization of the vaccine candidates according to the vaccine target. (Parts of the figure and the graphical abstract were created by using pictures from Servier Medical Art. Servier Medical Art by Servier is licensed under a Creative Commons Attribution 3.0 Unported License).

In total, 24 vaccines have been developed against RSV, and 2 of them are licensed for market use as preventive agents against lower respiratory tract infections. A substantial proportion of the vaccine candidates are aimed at the F protein of the RSV (Figure 1). A summary of the results is presented in Table 1. A PRISMA flowchart of the process of selecting the published articles of the clinical trials included in this review is available as a Supplementary Material [26]. 

### 3.1. mRNA Vaccines

mRNA as a vaccine platform carries genetic information exclusively for specific proteins, providing a higher safety grade due to the low probability of interference and modification of the genetic material of the host [74]. However, the likelihood of providing repeated doses to maintain immunity raises safety and efficiency issues regarding lipid aggregation [75,76]. Previous research in the mRNA technology field has significantly contributed to the rapid manufacturing process of the vaccines against SARS-CoV-2 [77]. The contribution of mRNA technology can be crucial to the development of vaccines in outbreaks of infectious diseases as well as in cancer treatments [78,79].

#### 3.1.1. mRNA-1345

mRNA-1345 is a vaccine candidate currently in Phase III of development, manufactured by Moderna as an improved candidate of Merck’s mRNA-1777 vaccine, aiming to achieve a stabilized prefusion form of the F (preF) RSV protein [20]. The interim results of a Phase I trial (NCT04528719) of the candidate showed acceptable safety characteristics in younger adults and raised neutralizing antibody titers against both strains of RSV [27,28]. Women of child-bearing potential and children aged between 12 and 59 months were also tested in this study. ConquerRSV (NCT05127434) is an ongoing Phase II/III vaccine study in adults above 60 years of age. In February 2022, the company proceeded to the Phase III part after the preliminary review of the Phase II data, showing a vaccine efficacy of 82.4% for RSV-LRTD defined with ≥3 symptoms and 83.7% for RSV-LRTD defined with ≥2 symptoms with adequate safety analysis [80]. Supported by these results, the market approval of mRNA-1345 was requested by the company in July 2023 and addressed to EMA, Swissmedic, and Therapeutic Goods Administration (TGA) as a preventive medicine against LRTD and acute respiratory disease (ARD) related to RSV in an adult population ≥ 60 years of age. A Biologics License Application (BLA) to the FDA is also running [81]. ROSE (NCT05572658) is an additional prospective study based on data from the ConquerRSV aiming to assess the impact on the healthcare system and the economy. Another ongoing Phase III trial (NCT05330975) named RSVictory is testing the co-administration of mRNA-1345 with a seasonal quadrivalent influenza vaccine (Afluria Quadrivalent) or mRNA-1273.214 (against SARS-CoV-2). 

#### 3.1.2. RSV mRNA LNP CL-0059 or RSV mRNA LNP CL-0137

Sanofi recently initiated a Phase I/II clinical trial (NCT05639894) in adults of a new mRNA vaccine candidate delivered via one of two different types of lipid nanoparticles (LNPs), named LNP CL-0059 and LNP CL-0137, which is expected to end on April 2025. 

### 3.2. Live-Attenuated/Chimeric Vaccines (LAVs)

LAVs constitute an important milestone in the history of medicine. Edward Jenner’s observation regarding the smallpox field provided the impetus for the development of vaccination, and at present, a broad spectrum of LAVs is in clinical Phase IV of development [82]. The deletion of genetic information for specific proteins results in attenuated viruses or microbes promoting essential replication procedures within a living organism without being virulent. It has been proven that they induce higher efficacy rates regarding the immune responses relative to the other vaccine types without the obligatory addition of adjuvants by inducing an immune response similar to the response in the case of natural infection with the pathogen [83]. The vaccination scheme includes a single dose providing long-term immunity [84]. LAVs are generally avoided in immunocompromised people and pregnant women due to severe infection or congenital transmission risks, respectively [85]. Intranasally administered LAV RSV vaccines have not shown, to date, a correlation with enhanced RSV disease and can result in acceptable immunity induction, including mucosal immunity. The current vaccine studies show effective immunogenicity in RSV-naïve infants, although late-stage trials are still needed [86]. Reverse genetic methods have led to the ΔM2-2 deletion and ΔNS2 deletion of the genetic material of RSV. These modifications inactivate the viral replication and optimize the induction of innate immunity of the host, respectively. Chimeric vaccines include attenuated viruses of a related pathogen modified to express the specific genes of the virus of interest. Two chimeric vaccines that undergo development regarding RSV are the rBCG-N-hRSV and the SeV/RSV vaccine candidates [87,88,89]. 

#### 3.2.1. BLB-201

A parainfluenza virus type 5 (PIV5) encoding the full-length RSV F protein is used to deliver the antigen through the nasal route in the BLB-201 vaccine candidate. BLB-201 was developed by Blue Lake Biotechnology and intends to prevent viral infection in the older adult population and young children under 2 years of age; it is currently in Phase II of development. The positive interim data of a Phase I trial (NCT05281263) shared by the company indicated a rise in antibody responses in 64% of the participants and a low replication rate without major safety concerns in adult participants [56]. 

#### 3.2.2. CodaVax-RSV

CodaVax-RSV by Codagenix is based on hundreds of modifications in the genome that produce an attenuated, completely functional in terms of antigen production virus that is not virulent. The data from preclinical studies indicate that the virus was significantly less infectious than the wild-type of RSV because the process of new virion production in the host is decelerated and, at the same time, the induction of cellular and humoral immunity that provides protection after an RSV challenge is achieved [57]. A Phase I study (NCT04295070) was completed and reached the primary endpoint regarding the safety profile and induced cellular immunity, according to an announcement from the company. In addition, another Phase I study is planned to be completed in May 2024 [57,58].

#### 3.2.3. RSV-ΔG

In this Intravacc’s candidate, the genetic information for the G protein is deleted from the virus, as it is hypothesized that RSV virions without the G protein can still activate immunity while being inactive. Cotton rat models showed the safety and immunogenicity characteristics of the modified virus without the G protein through intranasal administration [90]. The results of the Phase I trial did not show a significant induction of immunity in the seropositive population that was tested, and as a result, a dose-escalation trial with seropositive and seronegative children is the next step of the company [59]. 

#### 3.2.4. rBCG-N-hRSV

The Mycobacterium bovis bacillus Calmette–Guérin (BCG) is in clinical practice and has been proven effective in the induction of immunity. These characteristics, in combination with the fact that BCG can be safely administered in young children and infants, make BCG a suitable candidate for use as a recombinant vaccine vector. rBCG-N-hRSV, a candidate developed from Pontificia Universidad Catolica de Chile, consists of the live-attenuated BCG vaccine modified to express the N protein of RSV. The preclinical studies of recombinant BCG carrying genetic information for the N or M2 proteins of RSV in mice have indicated high effectivity rates [91,92]. Furthermore, this vaccine formulation was safe and capable of inducing protective immunity in cattle as a newborn animal model. Vaccination with rBCG-N-hRSV triggered a humoral and cellular response that protected cattle against bovine RSV, which is a natural pathogen for these animals [93]. The first human Phase I clinical trial (NCT03213405) revealed a safe candidate with no virulence evidence and increasing trends in IgG antibody values against both N-RSV and BCG (anti-PPD), with suggestions of further testing of the candidate in children and elderly [60]. To date, rBCG-N-hRSV is the only formulation that could be considered for use in newborns, particularly in countries where BCG is currently used as a vaccine for tuberculosis.

#### 3.2.5. SeVRSV

SeVRSV, supported by the National Institute of Allergy and Infectious Disease, expresses genetic information for the production of the F Protein from RSV and immunizes infants against human PIV-1 and RSV based on the Sendai virus, which is a parainfluenza virus-type 1 (PIV-1) that is not virulent in humans [61,94]. Despite the low levels of induced immune response in a group of seropositive adults tested in a Phase I clinical trial (NCT03473002), the safety characteristics with mild or moderate AEs suggest the further evaluation of the vaccine in seronegative children [61]. 

#### 3.2.6. MV-012-968

MV-012-968, a candidate of Meissa vaccines, is a LAV produced with the codon deoptimization of the NS1, NS2, and G genes and SH gene remotion, resulting in modified unusual codons and leading to incompetent translation [62,95]. Therefore, a high attenuation rate can be achieved [62,95]. An open-label Phase I trial (NCT04227210) in adults confirmed the attenuation of the vaccine and mucosal RSV-specific immunity induction. However, RSV-specific preF antibody titers in serum were not increased due to the initial seropositivity. An increasing trend was observed in RSV-specific mucosal IgA titers [62,63]. Seropositive children tested in a Phase Ib trial (NCT04444284) experienced specific mucosal immunity induction with a good safety profile and no signs of viral replication [64]. The interim data of a Phase I trial (NCT04909021) including seronegative children confirmed its safety and indicated the promotion of neutralizing antibody immunity in 78% of the study group population with the rate increasing to 89%, including the mucosal response [65]. The Phase II trial (NCT04690335) of the vaccine has been completed with no available results. 

#### 3.2.7. VAD00001

Sanofi Pasteur recently completed a Phase I/II trial (NCT04491877) study of VAD00001, a LAV, in children without published results. No further information in terms of the attenuation method has been provided.

#### 3.2.8. RSV ΔNS2/Δ1313/I1314L

The attenuation technique of this candidate includes a combination of the deletion of the NS2 gene from the genome, which is an RSV IFN antagonist gene, the deletion of Δ1313 codon in the L gene, and a stabilizing modification at codon 1314 [96]. The National Institute of Allergy and Infectious Diseases (NIAID) supports the development of RSV ΔNS2/Δ1313/I1314L, RSV LID/ΔM2–2/1030s, RSV 6120/ΔNS2/1030s, and RSV 6120/F1/G2/ΔNS1/RSV 6120/ΔNS1. A Phase I clinical trial showed an attenuated yet highly infectious candidate [66]. RSV ΔNS2/Δ1313/I1314L was tested together with RSV 276, another vaccine candidate, in a Phase I trial (NCT03227029) in children. In general, the immunogenicity parameters for both vaccines showed great infectivity, even though RSV ΔNS2/Δ1313/I1314L showed lower rates than anticipated, possibly due to previous RSV exposure. Regarding tolerability, the only concern arose with RSV/276-associated cough cases. RSV ΔNS2/Δ1313/I1314L will be assessed in other clinical trials [67]. A Phase I/II study (NCT03916185) is ongoing at present. 

#### 3.2.9. RSV LID/ΔM2–2/1030s

This candidate is based on an attenuation technique that deletes the M2-2 protein. This deletion was previously tested with other vaccine candidates and induced antigen expression with low levels of viral replication. An additional temperature-sensitive mutation in the polymerase protein L (1030s) completes the structure description of the vaccine candidate [68]. The vaccine was shown to be safe and stable in a Phase I clinical trial (NCT02794870 and NCT02952339) in seronegative children, and an increase in serum antibody assays and RSV F-specific immunoglobulin G (IgG) antibody titers were reported in 90% and 85% of the vaccine recipients, respectively. 

#### 3.2.10. RSV 6120/ΔNS2/1030s

An NS2 deletion mutation characterizes this candidate. Additionally, the modification in the polymerase L protein (1030s), as tested in the previous candidate RSV LID/ΔM2–2/1030s, is part of this vaccine candidate [97]. Seropositive and seronegative children participated in a Phase I trial (NCT03387137) of this vaccine candidate without available results. 

#### 3.2.11. RSV 6120/F1/G2/ΔNS1/RSV 6120/ΔNS1

RSV 6120/ΔNS1 contains a deletion in the NS1. In comparison, RSV 6120/F1/G2/ΔNS1 is similar to the previous candidate modified with codon optimization regarding the F gene and transport of both the F and G genes in other genome positions to optimize the translation [97]. The Phase I (NCT03596801) trial that intends to evaluate the vaccines is currently recruiting pediatric participants.

### 3.3. Subunit/Virus-Like-Particle (VLP)-Based Vaccines

Subunit vaccines consist of purified fragments of the desired pathogen, which can be peptides, proteins, or polysaccharides, lacking the genome of the whole pathogen, resulting in a non-virulent vaccine with an increased level of vaccine safety [98]. The hepatitis B virus (HBV) vaccine was the first subunit vaccine available for evaluation in the market in 1986 [99]. A significant advantage of subunit vaccines is the high level of safety due to the lack of pathogenicity, making them an appropriate candidate for the immunization of immunosuppressed individuals [100]. At the same time, however, this specific characteristic may lead to reduced vaccine effectiveness and the need for adjuvant use [101]. An important limitation in the use of subunit RSV vaccines occurs in the pediatric population based on the results of the formalin-inactivated RSV vaccine. The researchers abandoned the plan of development of this class of vaccines for the pediatric population [102]. Booster dosages are usually needed to result in long-term immunity [103]. VLP vaccines are a subclass of subunit vaccines using virus-derived components that form a particle structure, which retains similarities to the parent virus without the ability of replication because they do not contain the whole viral genome [104,105]. This characteristic makes VLPs suitable for use in immunocompromised or older adults [106].

#### 3.3.1. IVX-A12

IVX-A12 is a bivalent candidate of Icosavax and consists of IVX-121, a candidate against RSV, and IVX-241, a candidate against human metapneumovirus (hMPV), aiming to prevent both infections. The vaccine technology is based on VLPs, specifically regarding the IVX-121. The VLP leads to a multivalent presentation of the RSV-F protein in its stable prefusion form. Positive feedback from the interim analysis of the Phase I study (NCT05664334) was shared by the company on May 2023 with no significant safety signals and increase in the geometric mean titers (GMTs) of neutralizing antibody titers against both RSV strains with a 4-fold and 3-fold geometric mean fold rise (GMFR) measured for RSV-A and RSV-B in all groups of seropositive participants. Similar results also emerged for hMPV measurements. The simultaneous administration of the vaccines did not seem to influence the effects of each [31]. In June 2023, the company initiated the Phase IIa (NCT05903183) clinical trial of the vaccine candidate in adult participants aged between 60 and 85 years. 

#### 3.3.2. V-306

V-306, funded by Virometix, is a self-assembling VLP candidate that presents on its surface multiple epitopes of the antigenic site II of the RSV F protein (FsII), which is the binding site of palivizumab (PVZ) and is common to both pre- and post-fusion F (postF) protein forms [32]. V-306 was tested in a Phase I clinical trial (NCT04519073) in 60 healthy women of 18–45 years of age. The vaccine was safe and did not show tolerability issues. Regarding epitope-specific FsII IgG antibodies, an increasing trend was recorded in the intermediate- and high-dose groups, with a minimal further increase after the second dose. PVZ-competing antibodies (PCA) increased in the intermediate- and high-dose groups, indicating a PVZ-like antibody response. However, the investigators believe improvements are needed to show better results in future studies [32].

#### 3.3.3. DPX-RSV(A)

DPX-RSV from Immunovaccine uses an oil-based system called DepoVax (DPX). The antigen of interest is a peptide found in the RSV-A SHe protein. The SH protein as a vaccine antigen platform for RSV is new [33]. In a Phase I trial (NCT02472548), the vaccine showed potency of antibody induction, as shown by the increase in SHe-specific antibody titers in the context of a safe vaccine candidate. 

#### 3.3.4. VN-0200

Developed by a Japanese company, this candidate uses VAGA-9001a as the antigen combined with MABH-9002b as an immunostimulator. No further biological information is provided regarding to the antigen target. At present, a Phase II clinical trial (NCT05547087) is active in Japan, but there are no results shared from the completed Phase I trial (NCT04914520).

#### 3.3.5. BARS13 (ADV110)

Advaccine’s BARS13 uses the RSV G protein as the antigen of interest, produced in a bacterial (E. coli) platform. Additionally, cyclosporine A (CsA) is the factor that changes the immune responses and the solvent for reconstituting the RSV-G of the vaccine, and aims to prevent both RSV-A and RSV-B strains [34,107]. This combination intended to stimulate regulatory T cells (Treg) and the release of interleukin-10 (IL-10) to eliminate the chance of vaccine-enhanced disease (VED) development and significantly prevented VED [108]. BARS13 was clinically assessed in a Phase I study (NCT04851977), revealing a safe candidate with an increase in the measurements of RSV-G-specific IgG antibodies, which remained until day 60 after vaccination. After the second administration, the IgG antibody titers were escalated, indicating that the two-dose schedule induces better antibody-mediated immunity. The results suggested that the induced T-cell response is controlled and VED is not a possible development [34,35]. The company proceeded to the Phase II (NCT04681833) study conducted in Australia with an expected completion date of March 2024. 

#### 3.3.6. DS-Cav1 (VRC-RSVRGP084-00-VP)

RSV preF vaccines are manufactured for administration in pregnant women and older adults [36]. DS-Cav1 is developed by the National Institute of Health and the National Institute of Allergy and Infectious Disease. It constitutes a protein subunit molecule, specifically the stable product of the preF conformation of the RSV F protein, which is produced through modifications [109,110,111,112,113]. Specifically, the design of disulfide (DS) and cavity-filling alterations (Cav1) contribute to stabilization to the prefusion form of the F protein [114]. Modifications of the parent DS-Cav1 are being developed and tested in preclinical models. These techniques aim to optimize the induced immune activation [115,116,117,118]. The results of the Phase I trial confirmed an elevation of the titers of neutralizing antibodies against both RSV strains. At the same time, a second vaccination does not add clinical effects on long-term immunity. In general, the vaccine had an acceptable safety profile with a long-term induction of immunity until week 44, overcoming an RSV season. The addition of aluminum hydroxide (AlOH) showed no significant changes in antibody induction, making the vaccine suitable for use during pregnancy [36].

#### 3.3.7. Arexvy/RSVPreF3 OA (GSK3844766A)

Arexvy is a single-dose subunit vaccine, developed by GSK, resulting from the conjugation of the RSV-F protein stabilized in its prefusion form and adjuvant system 01 (AS01E) as immunostimulator and was the first preventive vaccine licensed by the FDA against RSV-mediated lower respiratory tract disease (LRTD) in subjects >60 years of age in the United States for market use on May 2023. The decision was predicated on the primary data of the ongoing Phase III clinical trial, which assessed the participants according to three RSV seasons. The FDA imposed post-marketing pharmacovigilance studies regarding evaluating Guillain–Barré syndrome and acute disseminated encephalomyelitis (ADEM) risks [119]. Additionally, the company is willing to assess the cases of atrial fibrillation in these studies. The vaccine is expected to be administered to the target population before the 2023/24 RSV season [44,120]. In June 2023, after an accelerated assessment of Arexvy by the EMA, the vaccine was approved for market release in Europe [121]. Several clinical trials evaluating the vaccine have already been completed. A Phase III clinical trial is currently ongoing that intends to add information related to the assessment of the immunity induction up to 3 years after a single dose of the vaccine and assessing the effect of the re-administration of the vaccine using different vaccination schemes. The primary safety results indicate a well-tolerated vaccine with a reported case of Guillain–Barré syndrome associated with the vaccine, according to the study investigator. Regarding immunological responses, the induction of specific immunity was demonstrated through the increase in the GMTs of neutralizing antibodies against RSV-A and RSV-B and geometric mean concentrations (GMCs) of RSVPreF3-specific IgG until one month after the vaccination, with a decrease until the month 6. Another Phase III trial (NCT04886596) is currently examining the prophylactic ability of RSVPreF3 OA against RSV-LRTD in older adults and has already enrolled 26,665 individuals. The interim data show high vaccine efficacy rates and protection against RSV acute respiratory infection (ARI) and RSV-LRTD in adults aged 60 years or older, even in the case of a chronic stable disease. Atrial fibrillation was experienced in 13 vaccinees and 15 placebo recipients at the 6-month follow-up [41,42,43,44]. The company is currently conducting other Phase III clinical trials. RSVPreF3 presents antigenic sites crucial to the mediated response, and for this reason, it was selected as the antigenic part of the vaccine. The AS01 adjuvant was already associated with the activation of immunity, especially in older adults, and a compound of these two factors proceeded to further clinical evaluation as GSK3844766A [37]. The maternal vaccine was also under development and included the same antigen of RSVPreF3 without using the adjuvant part of the vaccine. In February 2022, the company shared the termination of enrolling and vaccinating participants in trials referring to the maternal RSV vaccine candidate in pregnant women under the guidance of the Independent Data Monitoring Committee, while the safety parameters of the conducted trials are examined [122,123]. It was demonstrated that the unadjuvanted vaccine in pregnant women was related to a very small increase in preterm births, while the placebo recipients did not show a similar effect [41]. Specifically, the rate of preterm births was 6.81% for the pregnant women receiving the vaccine and 4.95% for the placebo recipients. Arexvy is not indicated for the immunization of persons < 60 years of age [124].

#### 3.3.8. Abrysvo/RSVpreF

Abrysvo is a bivalent subunit vaccine candidate based on developing the prefusion F protein as a stable molecule in this preF formulation administered as a single-dose regimen. The vaccine was developed by Pfizer and received authorization from the FDA against RSV-mediated lower respiratory tract disease for subjects 60 years of age or older after the approval of GSK’s Arexvy in May 2023. The results of an ongoing Phase III clinical trial named RENOIR (RSV vaccine efficacy study in older adults immunized against RSV disease) guided the decision of the FDA [125]. The interim safety results of the Phase III RENOIR study indicated a possible safety signal due to the occurrence of Guillain–Barré syndrome as a serious AE (SAE). However, other studies did not confirm evidence of Guillain–Barré syndrome or signals for other immune-mediated demyelinating conditions. The FDA announced on September 2023 a pharmacovigilance plan, which will be followed by the company. Potential safety signals that will be tested are risks for Guillain–Barré syndrome (GBS), allergic reactions, supraventricular arrhythmias, hypertensive disorders of pregnancy (HDP), and preterm births. Immunocompromised pregnant women and older adults will be included in further studies [53,126] The vaccine was also under accelerated assessment from the EMA with a pending decision for the marketing authorization application [127]. In July 2023, the Committee for Medicinal Products for Human Use (CHMP) stated a positive response regarding Abrysvo’s licensure, including both adult and maternal vaccine forms [128]. Recently, by August 2023, the FDA added the further indication of vaccination with unadjuvanted Abrysvo during the third trimester of pregnancy, especially between the 32nd and 36th week of gestational age, contributing to the prevention of LRTD and severe disease caused by RSV in neonates and infants until the first 6 months after birth [129]. Subsequently, the EMA licensed Abrysvo in the European Market as an immunizing agent against LRTD in the age group of adults 60 years of age or older and during the 24th and 36th weeks of gestational age for maternal use to provide infant protection. Abrysvo is currently the only market-approved immunizing agent in the field of RSV prevention, aiming at the target group of infants [130]. As stated in the prescribing information of Abrysvo, a not statistically significant disproportionate incidence of preterm births occurred between vaccinees and placebo recipients. It is recommended to use the vaccine according to the indication, because a causality between vaccination and preterm birth cannot be excluded based on the existing data [131,132]. 

The technology based on the crystal structure of the F protein announced by the National Institutes of Health (NIH) [113] is used in this vaccine candidate. The antigens that compose the vaccine are equal to 60 μg of the preF protein from both RSV-A and RSV-B strains of the virus. Phase I and II studies are already completed, while Phase III clinical trials are ongoing at present. MATISSE (MATernal Immunization Study for Safety and Efficacy) is a Phase III study (NCT04424316) in female pregnant subjects between 24 and 36 weeks of gestation testing the prevention of medically attended-lower respiratory tract infection (MA-LRTI) in infants through maternal vaccination. The study includes 14,750 participants and is expected to end in November 2023. Interim data were shared by the company in November 2022 indicating the tolerability and safety of the vaccine. The efficacy analysis presented a rate of 81.8% protection against severe MA-LRTI for infants within the first 90 days after birth. In the 6-month period, the efficacy reached a percentage of 69.4%. Moreover, no safety concerns for pregnant women or infants arose from the pre-review of the safety results [50,51]. RENOIR (NCT05035212) aims to assess the protective effect of the RSVpreF and the immune activation and safety parameters after a single administration of RSVpreF. From the interim analysis in August 2022, a vaccine efficacy of 66.7% for LRTI-RSV assessed as two or more symptoms and 85.7% using a definition of three or more symptoms emerged. The vaccine efficacy was 62.1% for the prevention of RSV-ARI. The safety results from a part of the population revealed an acceptable profile for the older adult group. Atrial fibrillation counted 10 cases in the vaccinees group compared to 4 in the placebo recipients, indicating a disproportion of this AE. SAEs that seem to be linked to the vaccine include hypersensitivity manifestation, Guillain–Barré syndrome, and Miller Fisher syndrome. In total, 2 cases of Guillain–Barré syndrome were recorded in a vaccinated number of 19.942 participants [52,53,54]. A Phase III study initiated in May 2023 named MONET constitutes a master protocol that will assess RSVpreF vaccine in adults aged 18 years and older with increased risk for developing severe RSV-mediated disease. 

### 3.4. Recombinant Viral-Vector-Based Vaccines

The first attempt to integrate genes into a virus, specifically Simian virus 40, was published in 1972 [133]. In this type of vaccine, the vector plays the main role in the induced response of the organism [134]. During the last years of the pandemic, the research and use of vector-based vaccines were significantly developed. The production mechanism involves modifying the viruses by adding the desired genetic information. As a result, the gene is expressed, and the protein is produced using the virus as a delivery system [135]. Specifically, the modified vaccinia virus Ankara (MVA) and adenovirus are increasingly used. The first approved vaccine of this category uses the modified yellow fever (YF) virus against Japanese encephalitis [136]. The vector is a key factor in the development of the vaccine, and thus, a rapidly increased production can be achieved in shortened periods [134]. Replication-competent viral vectors activate both cellular and humoral types of immunity. After replication, the progeny virus can increase and multiply the effect of the vaccine, just like a natural infection [134]. In the case of replication-defective vectors, the viral vector promotes a single cycle of reproduction and antigen production, a characteristic associated with safety compared to previous vectors [134]. On the other hand, an increased vaccine dosage or prime-boost vaccination strategy may be needed [137]. Alphaviruses, Arenaviruses, Adenoviruses, and MVA are used in this technology [134].

#### 3.4.1. MVA-BN-RSV

MVA-BN is already licensed by the authorities as a smallpox vaccine used in the adult population and immunocompromised people. MVA-BN-RSV developed by Bavarian Nordic, targets both strains of the RSV and transfers genetic information for proteins F, G (for both subtypes A and B), N, and M2 [70]. Bavarian Nordic collaborated with Nuance Pharma, which is active mainly in China, for further study and development in the Chinese market, including a second study, separate from the main Phase III trial in this population. In this way, access to the prevention of RSV infection is accelerated, including the Chinese and Asian populations [138,139]. After the positive results of Phase I and II clinical trials regarding the safety and induction of immunity, the company initiated the currently ongoing Phase III trial named VANIR. 

#### 3.4.2. Ad26.RSV.preF

Janssen manufactured Ad26.RSV.preF as a vaccine candidate for preventing RSV in older adults and the pediatric population. The vaccine uses replication-defective Adenovirus 26 as a vector, modified to express the RSV F protein from the RSV-A2 strain in its stabilized preF form [140]. Several clinical trials evaluated Ad26.RSV.preF in adults. EVERGREEN is a multicenter Phase III clinical trial that intends to test the potential of an Ad26.RSV.preF-based vaccine in preventing the LRTD caused by RSV in adults. In March 2023, the pharmaceutical company announced the withdrawal of the vaccine development against RSV of the older adult population and the early termination of the EVERGREEN study. According to the report, the different orientation of the company in terms of investing in medicines that have a high relation of benefit to the population is the basis of this change [141]. Seropositive children of 12–24 months of age were included in Phase I/II clinical trials (NCT03303625) testing Ad26.RSV.preF and the immunogenicity data from the toddler group showed an augmentation of RSV A2-antibody titers and the prefusion and post-fusion forms of the F protein. The antibody titers were maintained at higher levels than the initial measurements until 6 months after vaccination. Cross-neutralizing immunity may occur since RSV-B antibodies were also increased in the pediatric group without the occurrence of unexpected or severe AEs. Another Phase I/IIa study (NCT03606512) included seronegative children of the same age and has available results in ClinicalTrials.gov. In this trial, the antibody titers measured were higher from the baseline on day 85 and remained at higher levels until the end of the first RSV season, with the occurrence of SAEs in 5% (1/20) of the vaccinees. 

## 4. Discussion

RSV is linked to 3.4 million hospitalizations globally, of which 175,000 in the United States concern young children of the age of <5 years [142]. Concerning global mortality, death events range between 95,000 and 150,000 individuals, with approximately 14,000 fatal events in the adult population in the United States [142]. The results of an expanded systematic analysis in 2019 researching the number of RSV-associated all-cause deaths in hospital environments revealed a global mortality rate of 1 in every 50 deaths in the age group of 0–60 months and 1 in every 28 deaths in the age group ranging between 28 days and 6 months [143]. RSV is a pathogen that increases medical care costs globally, emphasizing the further need for prevention methods [144]. A meta-analysis including cost studies from 2000 to 2017 in children of 5 years of age and younger demonstrated that the inpatient and outpatient management costs of RSV acute lower respiratory infection (ALRI) range between EUR 3.47–7.93 billion. Additional direct and indirect costs further increase the financial load [145]. Academic institutions, global organizations, and the Pharma industry, including the World Health Organization (WHO), GAVI (The Vaccine Alliance), and the Bill & Melinda Gates Foundation, actively support research in the field of RSV [18,146]. Many ongoing studies are investigating the epidemiology and cost burden of RSV.

After identifying RSV, prevention was endeavored by developing the formalin-inactivated RSV vaccine. However, this venture led to tragic consequences, directly due to the cause of enhanced respiratory disease in the majority of the participants and the report of two cases of death in the young children participating in the study, who were challenged with RSV after immunization, and indirectly because this failure delayed significantly the progress in the field of the RSV vaccine development [89]. 

Arexvy is the first drug for active immunization against RSV, which gained approval from the FDA for market use in the age group of older adults aged 60 years or more [147]. Post-marketing surveillance studies are expected to investigate the risk of linkage to atrial fibrillation, Guillain–Barré syndrome, and acute disseminated encephalomyelitis [148]. Following the approval of GSK’s Arexvy, Pfizer also entered the market with Abrysvo, which the FDA also licensed for the older adult population in the United States. The unadjuvanted maternal candidate of Pfizer seems potent to reduce the disease in infants based on the results of the completed and ongoing clinical trials. A favorable recommendation from the appropriate authority of the FDA was already promoted, and the positive opinion regarding licensure from the Priority Review (PDUFA action date) for the maternal candidate was published in August 2023. Three days after the FDA licensure, the European Commission proceeded to accept the market release of the vaccine, including both indications of preventing LRTD actively in older adults over 60 years of age and passively in newborns and infants after maternal administration during pregnancy. This decision marks the first vaccine approval against RSV with the indication referred to maternal immunization [129,130].

At present, two more vaccines are near market approval, being in Phase III of clinical development for the older adult population. MVA-BN-RSV by Bavarian Nordic received, in February 2022, Breakthrough Therapy Designation permission from the FDA for the induction of immunity against lower respiratory tract disease induced by RSV in older adults of ≥60 years of age and is included in the priority medicines (PRIME) scheme of the EMA for the same indication [149,150]. The Phase III VANIR study is expected to end in December 2024. Moderna’s mRNA-1345 is also in Phase III of development and secured Fast Track designation for adults from the FDA in August 2021 [151]. 

The epidemiology and global disease burden of RSV highlight the need of prevention methods against the background of hampered vaccine development, years after viral identification. Recently, two vaccines were approved for active immunization against RSV, and multiple vaccine candidates, including mRNA, subunit and particle-based, live-attenuated or chimeric vaccines, and recombinant-vector-based vaccines, are under development. Based on the positive results of the clinical trials, an expansion of the approved preventive RSV vaccines is expected in the upcoming years, with a variety of new vaccines entering the clinical phases of development.

## 5. Expert Opinion

Vaccination against RSV can provide protection to high-risk populations and the community considering the cost burden of the disease. At present, a plethora of real-world data will be generated following vaccination of population groups according to the indications of the approved vaccines. Pharmacovigilance studies are expected to accompany the release of vaccines for the safety evaluation and safety signal detection. Simultaneously, the field of prevention is expanding as new evidence of various candidates under clinical development emerges. Positive data from evolving studies will lead to the further evolution of vaccine development. Within the next years, a variety of immunizing agents is expected to be approved for market use, collecting data from current clinical studies. Co-administration studies with other vaccines have already been conducted for some of the candidates, and more are expected to investigate the possible interaction and alteration of immune responses. Beyfortus can reduce RSV-LRTI and contribute to the decrease in RSV-related hospitalizations and mortality in newborns and infants. It is an important passive immunization agent, providing protection for an entire RSV season.

**Figure 2:**
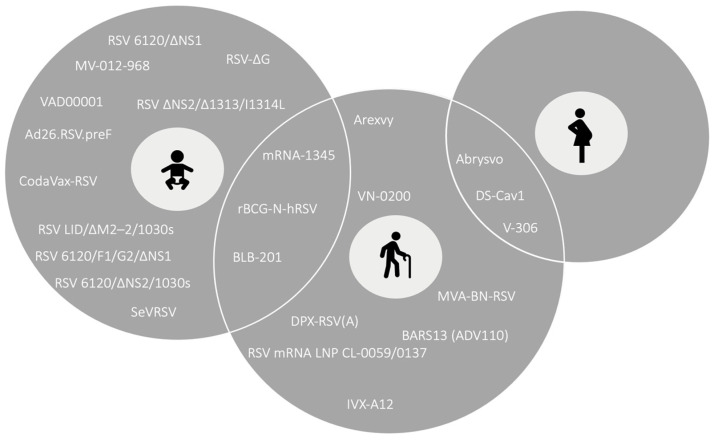
Age target groups of the vaccines.

The variety of advancing vaccines provides multiple advantages, since each demographic population is accompanied by specific characteristics influencing vaccine pharmacokinetics. For this reason, a single vaccine category cannot be used by all group populations susceptible to infection (Figure 2). Moreover, differences regarding the responses can also occur between individuals of the same demographic population based on the individual background of health, resulting in the coverage of different needs through the existence of multiple vaccines. Another important parameter regarding the establishment of prevention programs against RSV is the duration of the maintenance of induced immunity in real-world data. Epidemiological data from multiple studies can contribute to targeted prevention programs considering the outbreak periods of the virus by country. The determination of booster dose administration is also an important factor to produce effective immunity considering the various seasonality of the virus.

A significant aspect of the vaccination strategy is the availability of vaccines in low- and middle-income countries, where the incidence of RSV-related respiratory tract infections of a greater severity is significantly higher. The methods of delivery and storage of the vaccines in low- and middle-income countries should be determined in order to preserve the pharmacological properties of the immunizing agent. Simultaneously, choosing the appropriate characteristics of the vaccine agent based on the increased frequency of the specific comorbidities that may accompany these populations and the national health systems of these countries is important for the prevention of the disease in low- and middle-income countries. GAVI supports the investment of research in the field of RSV and impacts the vaccine supply in the countries included in GAVI’s strategies. 

The knowledge in the field of RSV is being improved as significant details in the pathophysiology of RSV infection in different age groups of patients are added. Various viral proteins are being investigated as possible target sites for vaccine candidates, including also new combinations of protein antigens and adjuvants to enhance the induction of immunity against both viral strains. Different vaccination development strategies target the vulnerable-specific groups of the population, being oriented to the challenges of each group. Understanding the special immune system characteristics of infancy is a challenge for the appropriate vaccine structure. Another significant limitation faced by researchers is the lack of defined limits of protective immunity levels. Public awareness, as shown during the SARS-CoV-2 pandemic, can also support the prevention of the disease and reduce total disease-associated mortality. Emphasizing information and pharmacovigilance mechanisms related to vaccine safety can contribute to further progress in the vaccination against RSV. The process of developing a vaccination strategy against RSV is characterized by complexity and special challenges; however, innovation in the field remains active at this point after the first vaccine approval.

## Figures and Tables

**Table 1:** Clinical trials and results of respiratory syncytial virus (RSV) vaccine candidates and market-approved vaccines.

Vaccine Category/ Vaccine Candidate	Vaccine Characteristics (Target Protein, Delivery System, and Adjuvant)	Target Population	Clinical Development Phase/ Clinical Trial Registration Number	Study Population	Results
**mRNA vaccines**					
**mRNA-1345**	RSV prefusion form (preF) of the F protein Lipid nanoparticles (LNPs) -	Older Adult Pediatric	Phase I		
			NCT04528719	Adults (18–49 and 65–79 years old), women of child-bearing potential (18–40 years old), and children (12–59 months old)	Interim analysis. No safety concerns. At least 20-fold and 11-fold increases in neutralizing antibody titers against RSV-A and RSV-B, respectively, for the younger adults and 14-fold and 10-fold increases for the older adults, respectively [27,28].
			NCT05397223	Adults (18–75 years old)	Ongoing.
			NCT05743881	Children (5-<24 months old)	Ongoing.
			NCT05585632	Adults (50–75 years old)	Ongoing.
			Phase II/III		
			ConquerRSV/NCT05127434	Adults (>60 years old)	Interim analysis. No safety concerns. Report of 9/64 events of RSV lower respiratory tract disease (LRTD) with ≥2 symptoms and 3/20 events with ≥3 symptoms for the vaccinees. Vaccine efficacy (VE): 83.7% for the definition of ≥2 symptoms and 82.4% for the definition of ≥3 symptoms [29,30].
			Phase III		
			RSVictory/NCT05330975	Adults (≥50 years old)	Ongoing.
**RSV mRNA LNP CL-0059** **RSV mRNA LNP CL-0137**	- LNPs (LNP CL-0059 or LNP CL-0137) -	Older adult	Phase I/II		
			NCT05639894	Adults, non-pregnant or in breastfeeding women (18–50, ≥60 years old)	Ongoing.
**Subunit/Viral-like-particle (VLP)-based vaccines**					
**IVX-A12 (VLP-based bivalent vaccine: IVX-121 against RSV and IVX-241 against hMPV)**	Stabilized RSV preF protein VLP MF59 (oil-in-water emulsion adjuvant) or none	Older adult	Phase I		
			NCT05664334	Adults (60–75 years old)	Interim analysis (May 2023). No safety concerns with comparable rates of adverse events (AEs) between vaccinees and placebo recipients. Induction of neutralizing antibodies against both strains of RSV and human metapneumovirus (hMPV), 4-fold and 3-fold geometric mean fold rise (GMFR) for RSV-A and RSV-B in seropositive participants, respectively; the combination is applicable [31].
			Phase II		
			NCT05903183	Adults (60–85 years old)	Ongoing.
**V-306 (synthetic VLP-based vaccine)**	Antigenic site II of RSV F protein (FsII) (preF and post-fusion form of the F protein (postF)) Self-assembling VLP -	Maternal Older adult (as a repeat dose stated from preclinical studies)	Phase I		
			NCT04519073	Non-pregnant women (18–45 years old)	Well-tolerated, transient AEs of mild or moderate severity, no serious AEs (SAEs) or vaccine-associated AEs of special interest (AESIs). FsIIm-specific immunoglobulin G (IgG) antibodies multiplied in the intermediate and high dose groups, RSV-A-neutralizing antibodies (nAbs), and RSV F-specific IgG Abs were not affected; a marginal increase in nAbs against RSV in some participants; improvement needed [32].
**DPX-RSV(A)**	SHe protein DepoVax(DPX) oil-based delivery system None or aluminum hydroxide	Older adult	Phase I		
			NCT02472548	Adults (≥50–64 years old)	No SAEs; no safety concerns; mostly mild or moderate severity of AEs. Geometric mean titer (GMT) rise in SHe-specific antibodies for the unadjuvanted vaccine regimen; maintenance of the response for up to 180 days [33]. Last update: 2020.
**VN-0200**	VAGA-9001a (no further information) - MABH-9002b (no further information)	Older adult	Phase I		
			NCT04914520	Japanese adults (≥20–≤50 years old and ≥65–≤80 years old)	Not available.
			Phase II		
			NCT05547087	Japanese adults (60–80 years)	Ongoing.
**BARS13 (ADV110)**	RSV G protein - None or cyclosporine A (CsA)	Older adult	Phase I		
			NCT04851977	Adults, including non-pregnant women who apply contraceptive measures (18–45 years old)	Safe; most AEs recorded were of mild severity with no SAEs. Increase in geometric mean concentration (GMC) and geometric mean fold increase (GMFI) in RSV G-specific IgG Abs and GMCs of nAbs after each dose remaining until day 60 and supporting a two-dose vaccine schedule. Post hoc analysis suggested that vaccine-enhanced disease (VED) development seems unlikely [34,35].
			Phase II		
			NCT04681833	Older adults (60–80 years old)	Ongoing.
**DS-Cav1 (VRC-RSVRGP084-00-VP)**	Stabilized RSV preF protein - None or aluminum hydroxide	Older adult Maternal	Phase I		
			NCT03049488	Adults (18–50 years old)	Well-tolerated with no SAEs and AEs of mild or moderate severity among groups. Multiple Ab inductions. nAbs against RSV-A and RSV-B increased, specifically for the high-dose group, and remained until week 44. IgG and immunoglobulin A (IgA) Abs against RSV-A preF, apex-binding, and side-binding epitopes also increased. A two-dose vaccine schedule and alum addition are not recommended [36].
**RSVPreF3 OA (GSK3844766A)** **Market-approved** **Name: Arexvy/AREXVY**	Stabilized in trimeric form RSV preF protein - Adjuvant System 01 (AS01E, AS01B)	Older adult Maternal (terminated on February 2022 due to safety signal -association with preterm births)	Phases I/II		
			NCT03814590	Adults (18–40 and 60–80 years old)	Well-tolerated. RSV-A and RSV-B nAbs increased and remained above baseline until month 14. The highest dose (120μg) and AS01E were chosen for further testing [37].
			Phase I		
			NCT04090658	Japanese adults (60–80 years old)	Well-tolerated. No SAEs associated with the vaccine. Increasing trend of RSVPreF3-specific IgG GMCs and neutralizing antibodies The two-dose vaccine schedule did not cause further increases [38].
			Phase II		
			NCT04657198 (extension trial of NCT03814590)	Older adult participants of NCT03814590	Low rates of SAEs. Similar recorded rates of pain as solicited local AE between groups, higher reported rate of unsolicited AEs, and similar reported rates for low- and medium-dose groups compared to the high-dose group. Humoral immunity activation observed by antibody titers’ increases.
			Phase III		
			NCT04732871	Older adults (≥60 years old)	Interim analysis. Well-tolerated. One vaccine-associated case of Guillain-Barré syndrome. Increase in RSV-A and RSV-B nAb GMTs and RSVPreF3-specific IgG GMCs until one month and maintenance at higher levels than the baseline until month 6 [39,40].
			NCT04841577 (co-administration with FLU-QIV)	Older adults (≥60 years old)	Two cases of acute disseminated encephalomyelitis (ADME), one of which was fatal, in the simultaneous co-administration group [41].
			NCT04886596	Older adults (≥60 years old)	VE against RSV-LRTD: 82.6%. VE against severe RSV-LRTD: 94.1% VE against RSV-acute respiratory infection (ARI): 71.7%. VE for participants with pre-existing stable diseases and the age group of 70–79 years of this category: 94.6% and 93.8%, respectively. Similar response among RSV-A and RSV-B. Solicited AEs of mild or moderate severity were common. Similar rates of SAEs, potential immune-mediated diseases (pIMDs), and fatal AEs between the groups. Severe-grade atrial fibrillation experienced by 13 vaccinees and 15 placebo recipients [41,42,43,44].
			NCT05059301	Older adults (≥60 years old)	Different lots of the vaccine caused similar antibody responses and similar rates of solicited local, systemic, and unsolicited AEs.
			NCT05559476 (co-administration with FLU HD vaccine)	Older adults (≥65 years old)	Ongoing.
			NCT05568797 (co-administration with FLU aQIV vaccine)	Older adults (≥65 years old)	Ongoing.
			NCT05590403	Adults (50–59 and ≥60 years old)	Ongoing.
**RSVpreF** **Market-approved** **Name: Abrysvo/ABRYSVO**	Stabilized RSV preF protein of both strains - Aluminium hydroxide (Al(OH)_3_)	Older adult (Approved) Maternal (Approved)	Phase I/II		
			NCT03529773 (alone or co-administration with SIIV)	Adults (18–45 and 50–85 years old)	No SAEs were vaccine-associated. GMTs of RSV-A and RSV-B nAbs increased with a more manifest increase in women and remained at levels above the baseline until 12 months for the younger and older adults. Younger adults experienced more common AEs compared to the older adults. The 120μg unadjuvanted regimen was selected for further assessment [45].
			NCT03572062 (test of CpG a TLR-9 receptor agonist as adjuvant)	Older adults (65–85 years old)	The study was halted since CpG did not increase immunogenicity [46].
			NCT05788237 (Phase Ib, co-administration with modRNA qIRV flu vaccine)	Older adults (≥60 years old)	Ongoing.
			Phase II		
			NCT04071158 (Phase IIb, co-administration with Tetanus, Diphtheria and Pertussis (TDAP))	Non-pregnant women (18–49 years old)	Mild/moderate AEs with similar distribution among groups; no safety concerns. Non-inferiority proof was documented for all the components of the vaccines, except for pertussis for the co-administration compared to the single administration of RSVpreF and TDAP [47].
			NCT04032093 (Phase IIb)	Pregnant women (18–49 years old)	Interim analysis. Similar or higher increase in the GMTs of RSV-A- and RSV-B-neutralizing antibodies to the non pregnant vaccinees of the previous trial; increased infant titers; no significant safety concerns for pregnant women or infants occurred; preterm births were measured at a rate of 3.7%. Post-hoc analysis showed a VE of 84.7% and 91.5% for medically attended RSV lower respiratory tract infection (MA-RSV-LRTI) and severe RSV lower respiratory tract infection (LRTI), respectively. The 120 μg without adjuvant proceeded to further evaluation [48].
			NCT04785612 (Phase IIa, RSV challenge study)	Adults (18–50 years old)	Vaccine was deemed safe and protective against RSV challenge; 6% of the vaccinees and 48% of the placebo recipients had symptoms during infection. Low levels of viral load measurements and shorter viral shedding for vaccinees. RSV-A and RSV-B nAb titers showed a 20-fold increase, and RSV preF-IgG titers increased. AEs had a similar pattern of report between groups and were of mild severity with no SAEs [49].
			Phase III		
			NCT04424316 (name: MATISSE)	Pregnant women (≤49 years old)	Interim analysis. Well-tolerated. No safety concerns for pregnant women or infants. VE: 81.8% protection against severe MA-LRTI for infants within 90 days after birth and 69.4% in the 6-month period. VE: 57.1% (not significant) clinically assessable for MA-LRTI and 51.3% in the 6-month period [50,51]
			NCT05035212 (name: RENOIR)	Older adults (≥60 years old)	Interim analysis VE: 66.7% for LRTI with ≥2 symptoms, 85.7% for LRTI with ≥3 symptoms, and 62.1% for RSV-ARI. Acceptable safety profile; fatal SAEs had a similar distribution among groups. AEs were common among vaccinees, especially in the age group of 60–69 years. Ten cases of atrial fibrillation occurred among the vaccine recipients compared to four in placebo recipients, and two cases of Guillain-Barré syndrome (possibly vaccine-associated) were recorded [52,53,54].
			NCT05096208 (tested 3 different lots)	Adults, including non-pregnant women (18–49 years old)	Well-tolerated, the primary outcome was met [53,55].
			NCT05301322 (co-administration with SIIV)	Older adults (≥65 years old)	Not available.
			NCT05842967 (name: MONET)	Adults with risk factors for severe RSV-mediated disease (≥18 years old)	Ongoing.
**Live-attenuated/Chimeric vaccines**					
**BLB-201**	Full-length RSV F-protein Live-attenuated parainfluenza virus-type 5 (PIV5) -	Pediatric (under 2 years of age) Older adult	Phase I		
			NCT05281263	Adults (18–59 years and 60–75 years old)	Interim analysis. A total of 64% of the vaccine recipients experienced a rise in RSV antibody responses and low replication rate, and there were no major safety concerns [56].
			Phases I/II		
			NCT05655182	Seropositive children (18–59 months old) and seropositive or seronegative children (6–24 months old)	Ongoing.
**CodaVax-RSV**	Modifications of the genome - -	Pediatric	Phase I		
			NCT04295070	Adults (18–49 and 50–75 years old)	Safe; induction of cellular immunity [57,58].
			NCT04919109	Seropositive (2–5 years old) and seronegative (6 months old to <2 years old) children	Ongoing
**RSV-** **Δ** **G**	Deletion of G-protein - -	Pediatric	Phase I		
			NTR7173 (Netherlands Trial Register)	Adults (18–50 years old)	AEs of mild or moderate severity with a similar distribution between groups; no SAEs; and no induction of immunity in the seropositive population. The next step is a trial including seronegative children [59].
**rBCG-N-hRSV (chimeric)**	Live-attenuated Mycobacterium bovis strain Bacillus-Calmette-Guérin (BCG) vaccine modified to express N protein of RSV	Pediatric Older adults	Phase I		
			NCT03213405	Younger adults (18–50 years old)	No evidence of virulence. AEs of mild or moderate severity. Dose-dependent increase trend for IgG antibodies against both N-RSV and BCG; the post-vaccination in vitro challenge of participants’ cells with N-protein activates the production of cytokines [60].
**SeVRSV (chimeric)**	Modified parainfluenza virus-type 1 (PIV-1) that expresses the F-protein of RSV	Pediatric	Phase I		
			NCT03473002	Adults (18–45 years old)	Frequently reported local and systemic AEs of mild or moderate severity; no SAEs. No 4-fold increase in RSV-specific antibodies (pre-exposed patients; information deficit). A further evaluation in seronegative children is planned [61].
**MV-012-968**	Codon deoptimization of NS1, NS2, and G genes and SH gene remotion for attenuation	Pediatric	Phase I		
			NCT04227210	Adults (18–40 years old)	Confirmed attenuation; mucosal RSV-specific immunity; RSV-specific antibody titers did not increase (seropositive population) [62,63].
			NCT04444284 (Phase Ib)	Seropositive children (15–59 months old)	Preliminary data. Specific mucosal immunity induction, good safety profile, and no signs of viral replication [64].
			NCT04909021 (Phase Ic)	Seronegative children (6–36 months old)	Interim data. Acceptable safety profile; mild solicited AEs; no SAEs. Changes in measurements of neutralizing antibody titers in 78% of the population with a rate of 89%, including the mucosal response [65].
			Phase II		
			NCT04690335 (viral challenge model)	Adults (18–45 years old)	Not available.
**VAD00001**	Live-attenuated	Pediatric	Phase I		
			NCT04491877	Children (6–18 months old)	Not available.
**RSV ΔNS2/Δ1313/I1314L**	Deletion of the NS2 gene, Δ1313 codon in the L gene, and stabilizing modification at codon 1314	Pediatric	Phase I		
			NCT01893554	Seropositive and seronegative children (4–59 months old)	Safe, highly infectious, and attenuated vaccine. Increase in RSV neutralizing antibodies and RSV F IgG antibody titers; no ≥4-fold increase in antibody titers in seropositive participants. A total of 2 of 15 and 4 of 20 children of the low- and high-dose group, respectively, experienced RSV-mediated medically attended acute respiratory infection (MAARIs). Anamnestic antibody response occurred [66].
			NCT03227029 (assessed also RSV 276, which resulted from a deletion of the M2-2 Protein)	Seronegative children (6–24 months old)	The acceptable safety profile for both vaccines presented, High reported ratio of cough cases with RSV/276; great infectivity for both vaccines; ≥4-fold increase in neutralizing antibody titers were measured in 60% of the RSV ΔNS2/Δ1313/I1314L recipients. Only RSV ΔNS2/Δ1313/I1314L was chosen to be further assessed [67].
			Phases I/II		
			NCT03916185 (assesses also RSV 6120/ΔNS2/1030s and RSV/276)	Seronegative children (6–24 months old)	Ongoing.
**RSV LID/ΔM2–2/1030s**	Attenuation based on deletion of the M2-2 protein and 1030s mutation at polymerase L protein	Pediatric	Phase I		
			NCT02794870 and NCT02952339	Seronegative children (6–24 months old)	No SAEs; mostly mild or moderate AEs. High infectivity rate; stable mutations; 4-fold increase in serum antibody assays in 90% of the vaccinees and ≥4-fold increase in RSV F-specific IgG antibody titers in 85% of the vaccinees. Anamnestic antibody induction [68].
			NCT04520659 (Phase Ib)	Seronegative children (6–24 months old)	Ongoing.
**RSV 6120/ΔNS2/1030s**	Attenuation based on the deletion of NS2 protein and 1030s modification of polymerase L protein	Pediatric	Phase I		
			NCT03387137	Seropositive (12–59 months old) and seronegative (6–24 months old) children	Infectious potential and immunogenicity were proven with greater rates of rhinorrhea in participants who received the candidate vaccine [69].
**RSV 6120/F1/G2/ΔNS1** **RSV 6120/ΔNS1**	Deletion of NS1 for both candidates and RSV 6120/F1/G2/ΔNS1 It is modified regarding the F gene, and F and G genes are transported in other genome positions	Pediatric	Phase I		
			NCT03596801	Seropositive (12–59 months old) and seronegative (6–24 months old) children	Ongoing.
**Recombinant vector-based vaccines**					
**MVA-BN-RSV**	MVA vector that transfers genetic information for Proteins F, G (both subtypes), N and M2	Older adult	Phase I		
			NCT02419391	Adults (18–49 years old and 50–65 years old)	No safety concerns. A 2-fold rise in interferon-γ (IFN-γ) GMTs. No increase in cells producing interleukin-4 (IL-4) (cellular immunity activation, protective factor against enhanced respiratory disease (ERD)). Increase to a lesser extent, in RSV-specific antibodies; similar responses between the age groups [70].
			Phase II		
			NCT02873286	Adults (≥55 years old)	High specific-antibody GMTs and until week 30 without the effect of a second dose. A 5- to 10-fold increase in T cell immunity titers against various RSV proteins; mucosal immunity is stimulated. All the high-dose group participants experienced immunity induction to, at least, one of three proteins and 65% to all proteins, with similar safety data compared to the previous Phase I trial with no safety concerns [71].
			NCT04752644 (challenge model)	Adults (18–50 years old)	Reduction in the viral burden in vaccinees; effective in inhibiting the onset of symptoms in RSV infection; similar safety data with regard to the previous trials [72].
			Phase III		
			NCT05238025 (name: VANIR)	Older adults (≥60 years old)	Ongoing
**Ad26.RSV.preF**	Replication of defective Adenovirus 26 as a vector modified to express stabilized pre-F from the RSV-A2 strain	Older adult—Discontinued (trials are not described) Pediatric	Phases I/II		
			NCT03303625	Younger adults (18–50 years old) and seropositive children (12–24 months old)	Safe in children and younger adults (as shown in previous studies in adult participants). High titers of RSV-A2-antibody titers and preF and postF antibodies after two vaccinations that remained until 6 months; RSV-B antibodies also increased in the pediatric group (cross-neutralizing immunity) [73].
			NCT03606512	Seronegative children (12–24 months old)	Antibody titers were higher from the baseline on day 85 and remained until the end of the first RSV season, with the occurrence of SAEs in 5% of the vaccinees.

## Data Availability

Data sharing is not applicable.

## Supplementary Materials

The following supporting information can be downloaded at: https://www.mdpi.com/article/10.3390/pathogens12101259/s1. Figure S1: Prisma flowchart presenting the process of selecting the published articles of the clinical trials.
