# Supplementary material for: Respiratory Syncytial Virus Vaccines: A Review of the Candidates and the Approved Vaccines
Source: Pathogens. 2023 Oct 19;12(10):1259. doi: 10.3390/pathogens12101259 (PMC10609699; doi:10.3390/pathogens12101259)
Supplement: Supplementary file 1 [file pathogens-12-01259-s001.zip › pathogens-2655473-supplementary.pdf]

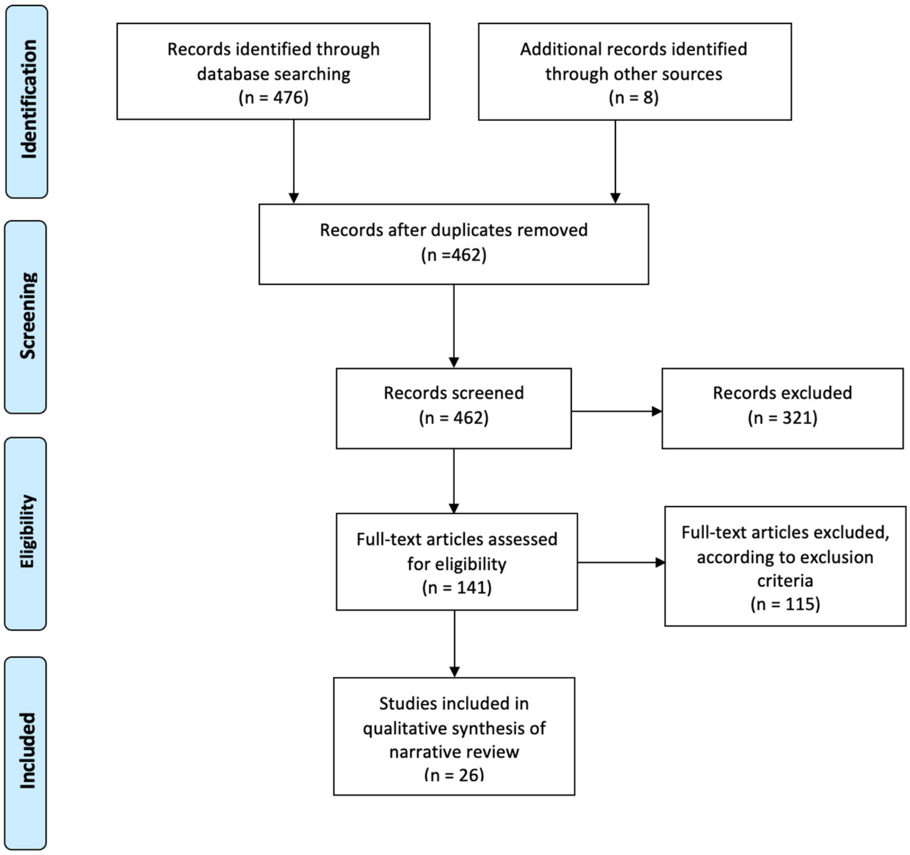

Figure S1. Prisma flowchart presenting the process of selecting the published articles of the clinical trials.
